# Effect of Mesenchymal Stem Cell-derived Microvesicles on Megakaryocytic Differentiation of CD34^+^ Hematopoietic Stem Cells

**DOI:** 10.34172/apb.2020.038

**Published:** 2020-02-20

**Authors:** Sara Aqmasheh, Karim Shamsasenjan, Elham Khalaf Adeli, Aliakbar Movassaghpourakbari, Parvin Akbarzadehlaleh, Davod Pashoutan Sarvar, Hamzeh Timari

**Affiliations:** ^1^Umbilical Cord Blood Stem Cell Research Center, Tabriz University of Medical Sciences, Tabriz, Iran.; ^2^Blood Transfusion Research Center, High Institute for Research and Education in Transfusion Medicine, Tehran, Iran.; ^3^Hematology and Oncology Research Center, Tabriz University of Medical Sciences, Tabriz, Iran.; ^4^Department of Pharmaceutical Biotechnology, Tabriz University of Medical Science, Tabriz, Iran.; ^5^Asadabad School of Medical Sciences, Asadabad, Iran.v

**Keywords:** Microvesicle, Hematopoietic stem/ progenitor cells, Mesenchymal stem cells, Megakaryocyte

## Abstract

***Purpose:*** Mesenchymal stem cells (MSCs) release hematopoietic cytokines, growth factors, and Microvesicles (MVs) supporting the hematopoietic stem cells (HSCs). MVs released from various cells, playing a crucial role in biological functions of their parental cells. MSC-derived MVs contain microRNAs and proteins with key roles in the regulation of hematopoiesis. Umbilical cord blood (UCB) is a source for transplantation but the long-term recovery of platelets is a main problem. Therefore, we intend to show that MSC-MVs are able to improve the differentiation of UCB-derived CD34^+^ cells to megakaryocyte lineage.

***Methods:*** In this descriptive study, MSCs were cultured in DMEM to collect the culture supernatant, which was ultracentrifuged for the isolation of MVs. HSCs were isolated from UCB using MACS method and cultured in IMDM supplemented with cytokines and MVs in three different conditions. Megakaryocyte differentiation was evaluated through the expression of specific markers and genes after 72 hours, and the data was analyzed by t test (*P*<0.05).

***Results:*** The expression of specific megakaryocyte markers (CD41 and CD61) in the presence of different concentrations of MSC-MVs did not show any significant difference. Also, the expression of specific genes of megakaryocyte lineage was compared with control group. The expression of GATA2 and c-Mpl was significantly increased, GATA1 was not significantly decreased, and FLI1 was significantly decreased.

***Conclusion:*** MSC-MVs could improve the expression of specific megakaryocyte genes; however, there was no significant expression of CD markers. Further studies, including the evaluation of late stages of megakaryocyte differentiation, are required to evaluate platelet production and shedding

## Introduction

Hematopoietic stem cell (HSC) transplantation has turned into an accepted procedure in the treatment of hematologic disorders.^1,2^ In comparison with bone marrow or mobilized peripheral blood progenitor cells, umbilical cord blood (UCB) has been considered as an attractive source of HSCs because of no need for high compatibility of HLA, easy collection of HSCs, and low incidence of graft versus host disease.^3-5^ However, a main limitation of UCB is the delayed hematopoietic engraftment, particularly platelet recovery,^6-8^ which restricts the use of UCB transplantation.^3^ Co-transplantation of *ex vivo* generated megakaryocytes with UCB-derived HSC can be a promising solution to reduce the lengthy period of platelet recovery.^9-11^ As an essential component of HSC niche, mesenchymal stem cells (MSCs) play a key role in hematopoiesis.^12^ They release a variety of hematopoiesis-regulating molecules and extracellular vesicles controlling functional characteristic of HSCs such as quiescence, self-renewal, and differentiation.^13-16^ Various studies have used MSCs as a feeder layer for the *ex vivo* expansion and differentiation of HSCs toward megakaryocytic progenitors.^14,17-19^ MSCs produce low levels of thrombopoietin (TPO), which synergizes with other cytokines such as IL-6, IL-11, and stem cell factor (SCF) for megakaryocyte differentiation in the absence of exogenous cytokines.^20,21^ Furthermore, MSCs secrete different types of membranous particles including exosomes, microvesicles (MVs), and apoptotic bodies into extracellular space.^22,23^ MVs are small and have a variety of sizes (100 nm-1 μm), containing lipid bilayer, mRNAs, miRNAs, siRNAs, surface markers, and cytokines originated from their parental cells.^24^ Various studies have surveyed biologic activities of MVs in intercellular communication network, self-renewal, and expansion of hematopoietic progenitor cells. Furthermore, the therapeutic potential of MSC-MVs for tissue injuries has been considered in several researches. Accumulated data have shown that MSC-MVs mimic biologic effects of their parental cells. Thus, we assumed that MSC-derived MVs may improve differentiation of HSCs toward megakaryocyte lineage similar to MSCs. In this study, we evaluated the synergistic effect of MSC-MVs and cytokines in the differentiation of UCB-derived CD34^+^ cells into megakaryocytes progenitor cells for achieving an effective strategy to improve megakaryocyte production.

## Materials and Methods

### Culture of UCB- derived MSCs

This study was approved by ethics committee of Tabriz University of Medical Sciences. UCB- derived MSCs were kindly donated by Dr. Nikougoftar Zarif (Blood Transfusion Research Center, High Institute for Research and Education in Transfusion Medicine, Tehran, Iran). MSCs were cultured in Dulbecco’s modified essential medium (DMEM; Gibco, USA) plus 10% heat-inactivated fetal bovine serum (FBS; Gibco, USA) and incubated under humidified incubator at 37℃ in a 5% CO_2_ atmosphere. When the monolayer of adherent cells reached 80% confluence, the supernatant was collected to extract MVs. The viability of cells was assessed by a dye exclusion assay by trypan blue staining.

### Isolation of MSC-derived MVs

Various methods have been introduced for the isolation of MVs from body fluids and conditioned cell cultures. We isolated MSC-derived MVs according to the following protocol.^25^ Briefly, MVs were purified from the supernatant of culture medium in several consecutive centrifugation steps using filtration tubes, which were followed by ultracentrifugation (Ultracentrifuge, Beckman, USA) (Beckman, UK). At first, to eliminate dead cells and cell debris, culture supernatant was centrifuged at 300 g for 10 minutes, at 2000×g for 10 minutes, and at 10 000 g for 30 minutes, respectively. Subsequently, centrifugal ultrafiltration (at 6000 g for 30 minutes) was used to concentrate the culture medium (100K nominal molecular weight limit filters; Beckman, UK).^26^ The supernatants from all stages were kept for the next step; the pellet was discarded and final supernatant ultracentrifuged at 100 000 g for 70 minutes. The pellet containing MVs was kept frozen but the supernatant was discarded.

### Characterization of MSC-derived MVs

#### Measuring the concentration of MSC- derived MVs

The concentration of MVs was determined using Bradford method.^27^ The Bradford assay is commercially available and rapidly presents an estimate of protein quantity, for which only 10 μL of each sample is required. For Bradford assay, samples containing MSC-MVs were thawed, 10 μL of MVs sample was diluted in 90 μL phosphate buffer saline (PBS, i.e., 1:10) on ice, and serial dilutions of samples were then prepared. Bradford solution (1000 μL) was poured into the microtubes; the sample was added to Bradford solution until its color was changed, and the volume reached 20 μL by distilled water. The concentrations were read in 595 nm by Bio Photometer (Pierce, Rockford, IL, USA) after 20 min of mixing, and MV-MSC concentration was measured up to 1:2 dilution.

#### Measuring the size of MSC-derived MVs

Dynamic light scattering (DLS) is an appropriate method to detect the size of MSC-MVs.^28-30^ This technology is frequently used in proteomic studies for the determination of particle size in a solution. DLS (Zetasizer Nano ZS, MALVERN, UK) uses the Brownian motions of MPs to evaluate the size and diffuse at a speed related to their size, with smaller particles diffusing faster than larger particles and the laser light scattering from mobile MPs being detected by avalanche photodiode detector. The intensity changes were analyzed by a digital auto correlator, which generated a correlation function. The suitable concentration was prepared as follows: 100 μL of MSC-MVs sample was diluted in 900 μL PBS in glass or quartz cells for size measurement and mean MPs size was read from the resultant graph.

### CD34^+^ cell purification

After obtaining informed consent, UCB samples were obtained from healthy pregnant women undergoing full term deliveries. First, UCB was diluted by phosphate buffer saline (PBS)/2mM EDTA and 0.5% FBS. Mononuclear cells (MNCs) were separated from UCB by Ficoll-Paque (1.077 g/cm^3^, GE Healthcare) and twice washed with PBS. The MNCs were enriched for positive selection of CD34^+^ antigen using magnetic activated cell sorting according to the manufacturer’s instructions (MACS; Miltenyi Biotec, Bergische Gladbach, Germany). The purity of UCB-derived CD34^+^ enriched cells was verified by flow cytometric analysis (FACSCalibur analyzer, BD Biosciences, USA) and counterstaining with phycoerythrin (PE)-conjugated anti-CD34 antibody (MoAb CD34- PE, DAKO- Glostrup, Denmark).

### Ex vivo expansion of CD34^+^ cells

Purified CD34^+^ cells were cultured in DMEM plus 10% FBS supplemented with recombinant cytokines, including SCF, Flt-3 ligand (FL), and TPO (100 ng/mL, all from stem cell technologies, Vancouver, BC, Canada).^31^ The cells were incubated under a fully humidified incubator at 37℃ with a 5% CO_2_ atmosphere for 7 days. On day 7, the count and viability of cells were detected by trypan blue dye exclusion test (Gibco, UK) using a Neubauer hemocytometer. Furthermore, the cells were evaluated by flow cytometry for verification of expansion without differentiation.

### Flow cytometric analysis

For immunophenotyping analysis, the cells were stained with monoclonal antibodies, including CD34-PE, CD41-PE, and CD61-PE (all from Dako, Glostrup, Denmark). Briefly, the cells collected from liquid cultures were washed and suspended in a final volume of 50 μL PBS. Then, 10 μL of each conjugated antibody (murine IgG1, Dako Cytomatin, Denmark) was added to sorting cells. The cells were incubated at 4°C for 20 minutes in a dark place. Following incubation, the cells were twice washed by PBS containing FBS (2%), then resuspended and fixed in cold paraformaldehyde (1%) at 4°C for 30 minutes. To establish nonspeciﬁc staining, appropriate conjugated mouse IgG1 isotype control antibodies were used, and flow cytometric analysis was performed (FACS Calibur, BD).

### Differentiation of expanded CD34^+^ cells to megakaryocyte progenitor cells

The expanded CD34^+^ cells were cultured in a Iscove’s modified Dulbecco’s medium (IMDM, Gibco, USA) supplemented with 10% FBS at a density of 100×10^3^ cells per well (200 μL) for 72 hours under three different conditions as follows: (a) The specific megakaryocyte lineage cytokine cocktail (CC), as a control medium supplemented with recombinant cytokines, including 25 ng/mL of SCF, FL, TPO, and IL-11, )b) CC plus MSC-MVs at 10 μg/mL concentrations, (C) CC plus MSC-MVs at 20 μg/mL concentrations. All culture media were incubated under a humidified incubator at 37°C with a 5% CO_2_ atmosphere. After 72 hours, the cells were evaluated for megakaryocyte differentiation using flow cytometric analysis.

### Gene expression analysis of GATA1, GATA2, FLI1 and TPO receptor (c-Mpl)

GATAs are members of a family of zinc finger transcription factors that are important in erythropoiesis and megakaryopoiesis. To evaluate megakaryocyte differentiation, the expression of specific genes, including *GATA1 (globin transcription factor-1), GATA2, FLI1 (Friend leukemia virus integration 1),* and *TPO receptor (c-Mpl)* was investigated after 72 hours. Firstly, total RNA was extracted using Trizol (Qiagen, Hilden, Germany). RNA concentration and quality were evaluated by absorbance ratio in OD 260/280 nm by spectrophotometric analysis (Picodrop, UK). cDNA synthesis was performed using the RevertAid™ First Strand cDNA Synthesis Kit and primers (Fermentas, Canada) according to the manufacturer’s instructions. The expression of *GATA1, GATA2, FLI1 (Friend leukemia virus integration 1),* and *TPO receptor (c-Mpl)* genes was tested by polymerase chain reaction (RT-PCR). During the process of amplification, the genes were added to 2X qPCR/RTD-PCR Master Mix E4 (SYBR Green AB kit) forward and reverse primers (Metabion, Germany), cDNA and double distilled water (ddH_2_O). Reactions were performed in Real-time PCR device (AB Applied Biosystems, Stephone Real-time PCR) and GAPDH gene was used as an internal control. Also, the expression level of two main cytokines, namely SDF-1 and GM-CSF, was measured in MSCs by qualitative PCR in normal condition. The pairs of primers used for gene amplification are presented in Table 1.

**Table 1:** Primers for real-time polymerase chain reaction (RT-PCR)

**Primer pair**	**Sequence (5'->3ʹ)**	**TM**	**Base pair**	**Product length**
GATA1-F	CACGACACTGTGGCGGAGAAAT	63	22	140
GATA1-R	TTCCAGATGCCTTGCGGTTTCG	64	22	140
GATA2-F	CAGCAAGGCTCGTTCCTGTTCA	63	22	150
GATA2-R	ATGAGTGGTCGGTTCTGCCCAT	64	22	150
FLI1-F	ACGGAAGTGCTGTTGTCACACC	63.5	22	140
FLI1-R	CAAGCTCCTCTTCTGACTGAGTC	60.5	23	140
c-Mpl-F	ACTCAGCGAGTCCTCTTTGTGG	62.3	22	154
c-Mpl-R	CATAGCGGAGTTCGTACCTCAG	60.3	22	154
GAPDH-F	ACCCATCACCATCTTCCAGGAG	61	22	159
GAPDH-R	GAAGGGGCGGAGATGATGAC	60	20	159

The primer pairs were designed for *GATA1, GATA2, FLI1, c-Mpl,* and *GAPDH* (as an internal control). The forward (F) and reverse (R) primers (Metabion, Germany) for gene amplification are presented in Table 1. For the amplification of genes, 2X qPCR/RTD-PCR Master Mix E4 (SYBR Green AB kit), ddH_2_O, forward (F) and reverse (R) primers, and cDNA were used. Amplification was performed in Real-time PCR device (AB Applied Biosystems, step one real-time PCR) and relative expression was assessed using ΔCT values normalized with the expression of endogenous reference gene (GAPDH mRNA) in a similar procedure.

### Statistical analysis

All data are shown as mean ± standard deviation (SD) and analyzed by GraphPad Prism v5.00 (GraphPad Software, San Diego, CA, USA). Statistical analysis was performed by student’s *t* test because data have been evaluated pairwise to show the effects of MSC-MVs and their concentrations on CD34^+^ to MK lineage and *P* < 0.05 was considered as significant.

## Results

### Characterization of MSC-MVs

**Figure 1:**
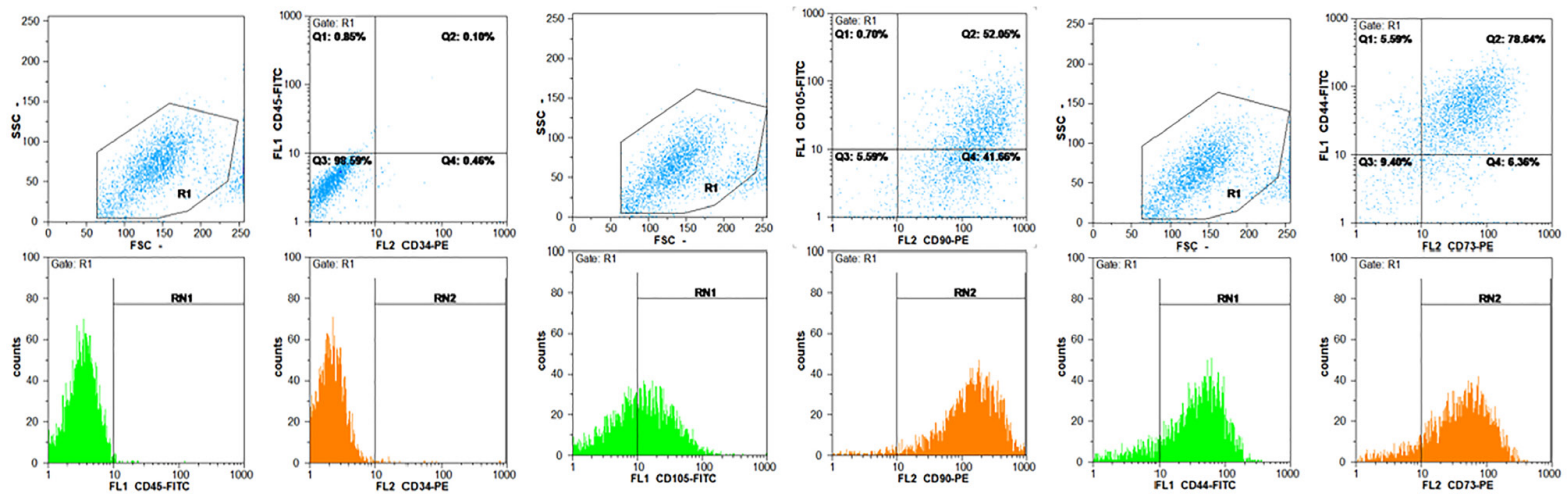
Results for CD34, CD45, CD44, CD105, CD90, and CD73. CD34 and CD45 are negative but CD44, CD105, CD90, and CD73 are positive; therefore, these cells are MSCs.

**Figure 2:**
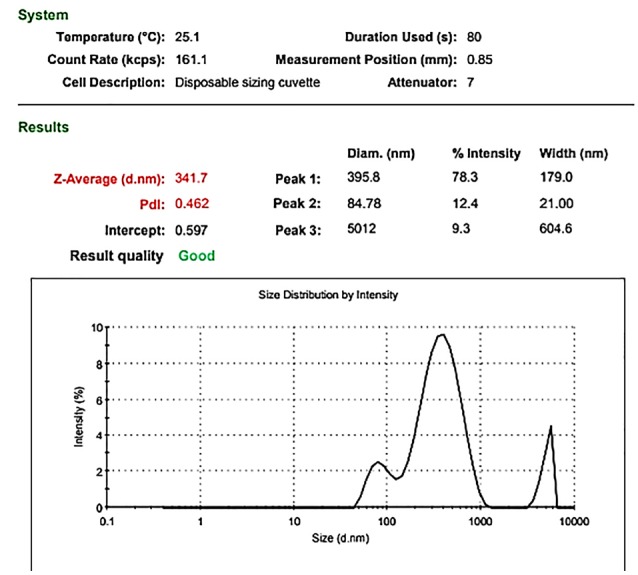
Size distribution report of MSC- derived MVs.

Flow cytometric results of MSCs showed that these cells were negative for CD34, CD45 but were positive for CD44, CD105, CD90, and CD73 (Figure 1). Then, the specifications of MSC-MVs were determined according to the method described in materials and methods section. The concentration of MSC-MVs was 171 μg/mL by Bradford assay. Furthermore, the size of MSC-MVs was determined as 341 nm by Nano particle analyzer using DLS technique (Figure 2).

### Expansion and differentiation UCB-CD34^+^ cells to megakaryocyte lineage

**Figure 3:**
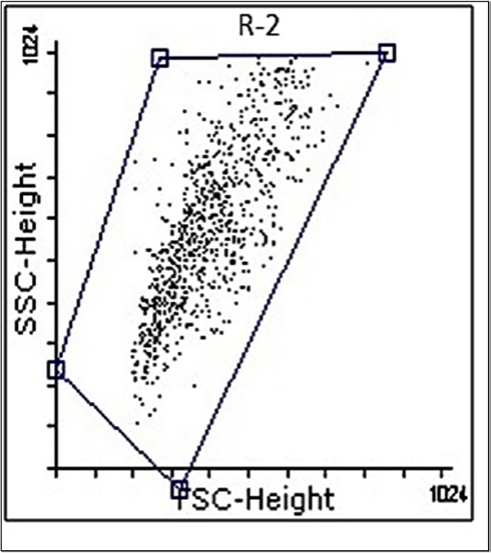
Gating of CD34^+^ cells.

**Figure 4:**
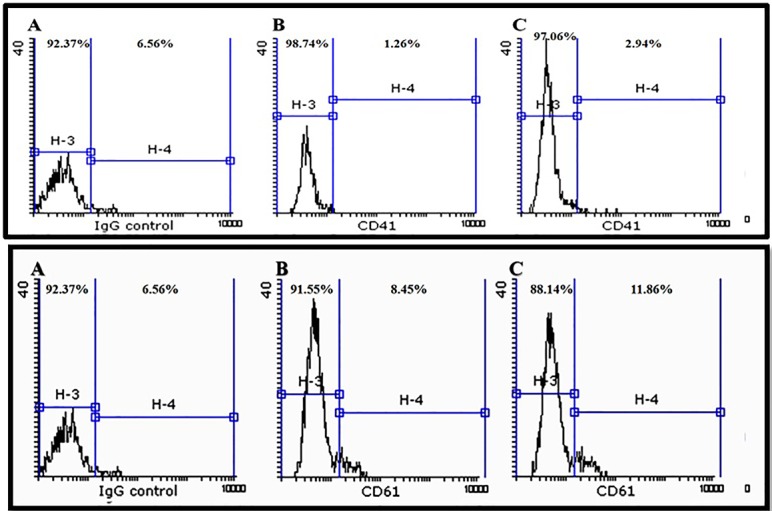
The expression of CD41 and CD61. Isotype-negative Control, (A) before expansion (B) after expansion (C).

**Figure 5:**
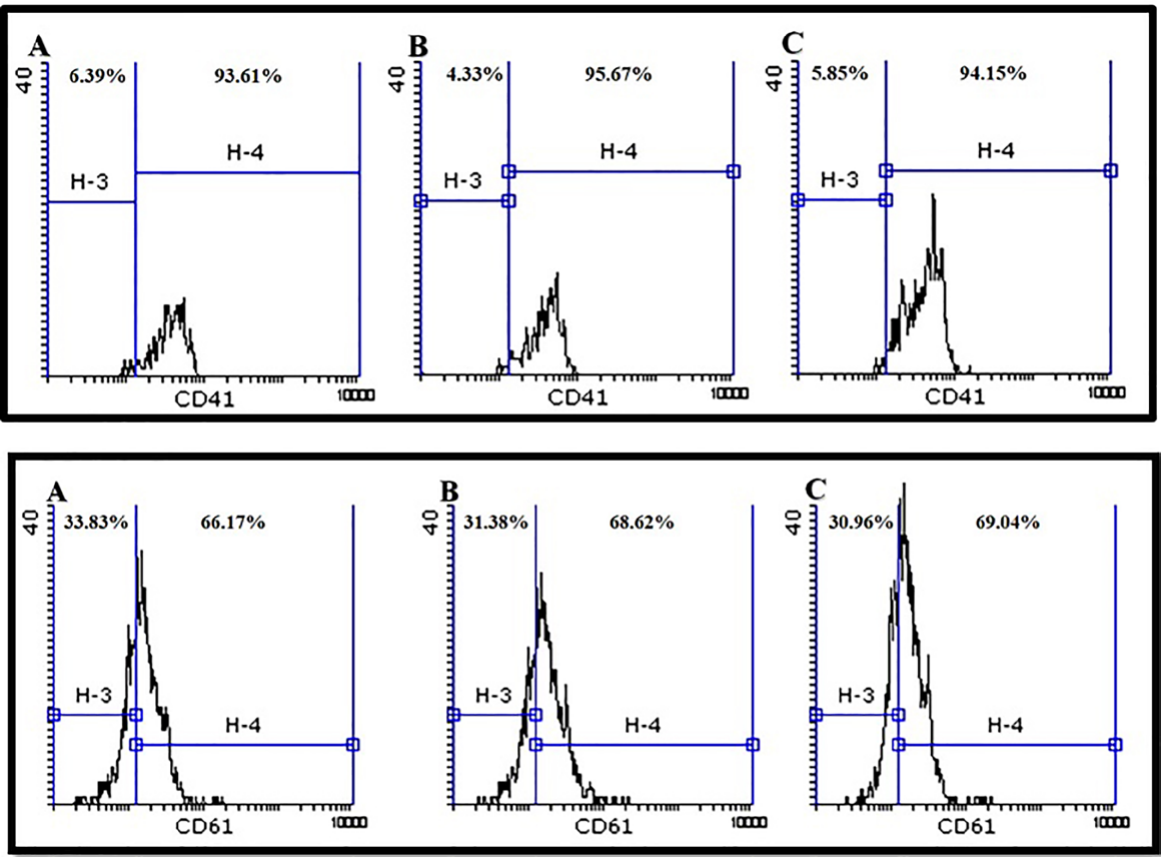
The expression of CD41 and CD61 markers in the presence of proliferation and differentiation cytokines (group-A) and 10 and 20 µg/mL concentrations of microvesicles (group-B and group-C, respectively).

The mean percentage of purified CD34^+^ cells was 92.56%±1.2 with 90%±1.5 viability (Figure 3). Furthermore, CD41 and CD61 were evaluated as markers for early megakaryocyte differentiation. Expression of CD41 and CD61 markers on expanded CD34^+^ cells was nearly 3% and 12%, respectively (Figure 4). We differentiated the expanded CD34^+^ cells toward megakaryocyte lineage for 72 hours under three conditions as described in materials and methods section. On day 3, expression of CD 41 was 95.67% and 94.15% in groups (a) and (b), respectively versus 94% in control group. Expression of CD 61 was 68.62% and 69.04% in groups (a) and (b), respectively versus 66.17% in control group. As shown in Figure 5, there was no significant difference in expression of either CD41 or CD61 in treated groups with the control group (*P* <  0.5).

### Effect of MSC-MVs treatment on the expression of megakaryocyte-specific genes

**Figure 6:**
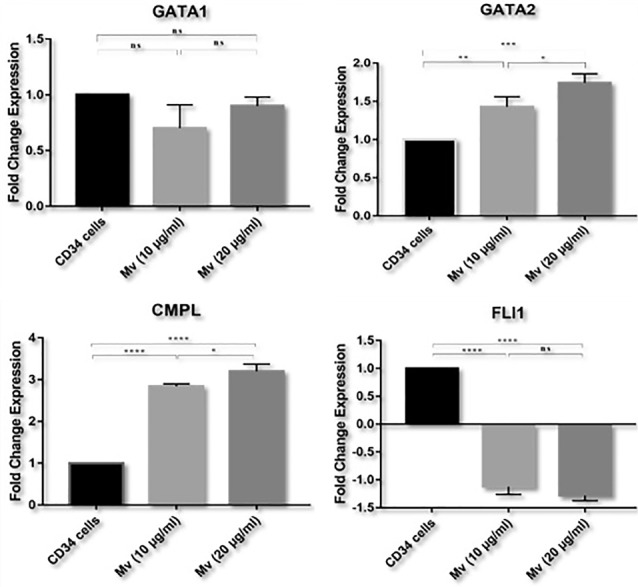
Relative quantification real-time PCR. Comparison results of GATA1, GATA2, FLI1, and c-Mpl expressions in three conditions: groups A, B, C. (*P* < 0.05) (*, **, ***, and **** represents 0.05 > 0.01 >0.001 >0.0001, respectively).

The expression of megakaryocyte-specific genes (GATA-1, GATA2, FLI1, and c-Mpl) was assessed by relative quantification real-time PCR. The gene expression in MVs-treated groups was compared with the control group. After normalization, the expression of GATA1 gene showed a decrease in group b and c in comparison with control group (group a), which was not a significant. The expression levels of GATA2 and c-Mpl genes showed a significant increase in comparison with control group. Moreover, the expression of FLI1 gene was significantly reduced relative to control group (*P* <  0.05). These results are shown in Figure 6.

## Discussion

A variety of biologic activities has been reported for MSC-MVs in intercellular communication network, drug delivery systems, and expansion of hematopoietic progenitor cells. Herewith, we focused on the supportive role of MSC-MVs in the differentiation of CD34^+^ UCB cells to megakaryocyte lineage. Our results showed that MSC-MVs induced an increase in the expression of specific genes of megakaryocyte lineage.

Delayed platelet recovery is a serious drawback after UCB-derived HSCs transplantation, which limits the use of UCB in transplantation.^32^ Many studies have been conducted with the aim of overcoming thrombocytopenia after UCB transplantation.^33^ The role of MSCs as an essential component in HSC niche has been shown in several studies,^13-16^ so that Hatami et al showed that co-culture of CD34^+^ UCB cells with MSCs under serum-free conditions in combination with specific megakaryocyte linage cytokines improved megakaryopoiesis.^19^ MSCs release large amounts of MVs to extracellular space, which contain mRNAs, miRNAs, siRNAs, surface markers, and cytokines originating from their parental cells.^34,35^ Supposing that MSC-MVs mimic the hematopoiesis effects of MSCs, Xie et al investigated effects of MSC-MVs on ex vivo expansion of UCB-derived HSCs and found that MSC-MVs increased proliferation and colonization of UCB-derived CD34^+^ cells.^16^ Pashoutan Sarvar et al reported that MSC-MVs suppressed differentiation of UCB-derived HSCs to erythroid lineage.^36^ To the best of our knowledge, the role of MSC-MVs in differentiation of CD34^+^ UCB cells to megakaryocyte lineage has not been studied. In the present study, we investigated the effect of MSC-MVs on the expression of megakaryocyte-specific genes and CD markers compared with the control group. Our data showed that MSC-MVs could promote the expression of megakaryocyte-specific genes in vitro, including GATA2 and c-MPL. GATA 2 is a transcription factor expressed in pluripotent stem cells that regulates megakaryopoiesis in early stages. TPO is known as an important megakaryocyte growth factor regulating megakaryocyte proliferation and maturation as well as platelet production via c-MPL receptor.^37^ In our research, the expression of GATA-1 was lower in treated groups compared with the control group; however, this decrease was not significant. As a transcription factor, GATA-1 plays a role in late cytoplasmic maturation, platelet biogenesis, and polyploidization.^38,39^ Moreover, FLI-1, which has a crucial role in polyploidization in late stages of megakaryopoiesis, showed significantly reduced expression.^40^

## Conclusion

Interestingly, the use of MSC-MVs in culture medium did not effect the expression of CD41 and CD61 as markers for early megakaryocyte differentiation. Furthermore, we did not find any significant difference in the expressions of specific genes and CD markers of megakaryocytes among the groups during megakaryocytes maturation. It is noteworthy that the expanded CD34^+^ cells were cultured with specific megakaryocyte lineage cytokines for a 3-day period, which is likely to have induced an increase in megakaryocyte-specific CD markers and FLI1 expression by increasing period of culture. In conclusion, many studies have been conducted with the aim of overcoming thrombocytopenia after UCB transplantation via expansion of megakaryocyte progenitor cells. However, the impact of MSC-MVs on differentiation of HSC to megakaryocyte lineage is not known. According to the finding of this study, we could not certainly conclude the effect of MSC-MVs on CD34^+^ cells differentiation to megakaryocyte lineage. Nevertheless, we need further investigations with longer incubation periods. The exact mechanism of MSC-MVs effect on hematopoiesis is unclear and further studies are required to investigate molecular mechanisms involved in regulatory effects of MSC-MVs on megakaryocyte differentiation.

## Ethical Issues

Not applicable.

## Conflict of Interest

Authors declare no conflict of interest in this study.

## Acknowledgments

This work was supported by the grant of Umbilical Cord Stem Cell Research Centre, Tabriz University of Medical Sciences. The authors appreciate Blood Transfusion Headquarter of East Azerbaijan Province for laboratory facilities, as well as our colleagues in Tabriz University of Medical Sciences, Tabriz, Iran and Pasteur institute, Tehran, Iran.
